# Visualising statistical models using dynamic nomograms

**DOI:** 10.1371/journal.pone.0225253

**Published:** 2019-11-15

**Authors:** Amirhossein Jalali, Alberto Alvarez-Iglesias, Davood Roshan, John Newell

**Affiliations:** 1 School of Mathematics, Statistics and Applied Mathematics, National University of Ireland, Galway, Ireland; 2 HRB Clinical Research Facility, National University of Ireland, Galway, Ireland; 3 CÚRAM, SFI Research Centre for Medical Devices, National University of Ireland, Galway, Ireland; University of Edinburgh, UNITED KINGDOM

## Abstract

Translational Statistics proposes to promote the use of Statistics within research and improve the communication of statistical findings in an accurate and accessible manner to diverse audiences. When statistical models become more complex, it becomes harder to evaluate the role of explanatory variables on the response. For example, the interpretation and communication of the effect of predictors in regression models where interactions or smoothing splines are included can be challenging. Informative graphical representations of statistical models play a critical translational role; static nomograms are one such useful tool to visualise statistical models. In this paper, we propose the use of dynamic nomogram as a translational tool which can accommodate models of increased complexity. In theory, all models appearing in the literature could be accompanied by the corresponding dynamic nomogram to translate models in an informative manner. The R package presented will facilitate this communication for a variety of linear and non-linear models.

## Introduction

Translational Medicine, within biomedical and public health research domains, is defined as the convergence of basic and clinical research with the aim to transfer knowledge on the benefits and risks of therapies. The concept of ‘Translational Statistics’ was proposed [1] to facilitate the integration of biostatistics within clinical research in order to enhance communication of statistical research findings in an accurate and accessible manner to diverse audiences (e.g. policy makers, patients and the media).

The use of appropriate graphics is central to all areas of statistical research. For example, the graphical representation of data is necessary to provide a way of assessing at least parts of any assumed statistical model before engaging in formal analysis and to aid in the presentation and understanding of results and conclusions. Nomograms have been used to visualise statistical models, playing the role of a graphical ‘predict’ function, facilitating the calculation of a point estimate of the response variable for a particular set of values of the explanatory variables. In this paper, we propose the use of dynamic nomograms as a visualisation and translational tool to further aid the communication of the results of a statistical analysis to a non-statistical audience. We introduce the **R** package DynNom for generating dynamic nomograms (as Shiny objects) for a variety of linear and non-linear models. In theory, most models presented in the literature could have an accompanying web address to direct the reader to the corresponding dynamic nomogram allowing them to ‘interact’ with the model to gain insight into the effect of each explanatory variable on the primary response of interest.

## Nomograms

**Fig 1:**
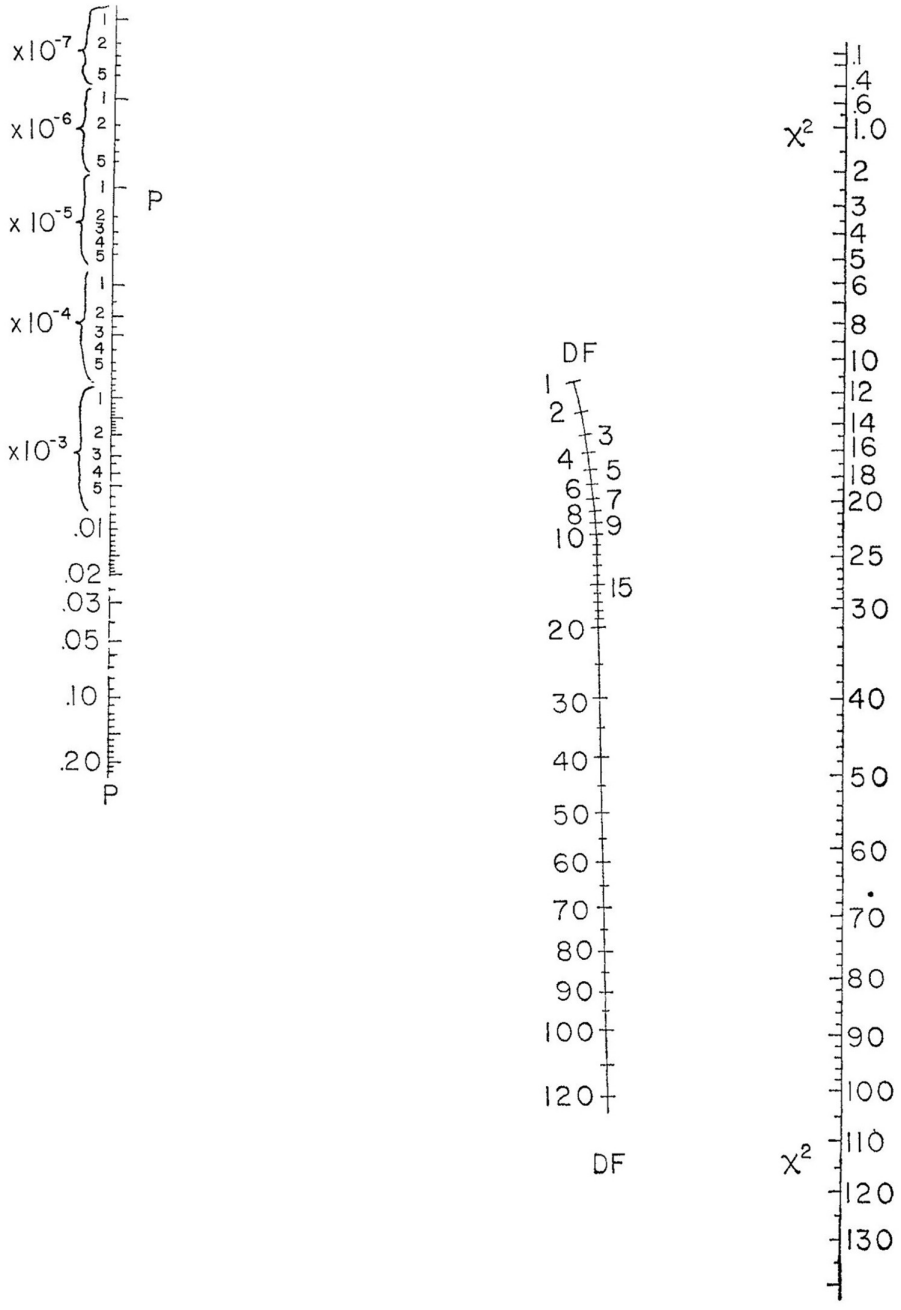
A nomogram to calculate the p-value for a chi-square test statistic [10]. To use the nomogram draw a straight line from the value of the chi-square test statistic through the required degrees to read off the corresponding p-value.

The field of nomography was invented by the French mathematician Maurice d’Ocagne in 1880 to provide engineers with fast graphical calculation tools for complicated formulas to a practical level of precision [2, 3]. An early use of nomograms is attributed to [4], where they were employed in mathematics for calculating elementary arithmetic, quadratic equations and trigonometrical functions. One of the first nomograms in statistics was used to calculate the coefficient of variation, interval estimates for a mean and the comparison of two means and variances [5]. Other examples of nomograms in this domain are the chi-square test nomogram (Fig 1), Altman’s nomogram to calculate sample size or power [6] and Fagan’s nomogram [7] for the applications of Bayes’s theorem. Fagan’s nomogram, widely used in the context of diagnostic tests, was recently reproduced as the Held’s nomogram for the calculation of post-test probabilities [8, 9].

Nomograms have been widely used in applications in science, engineering, the military, clinical research and epidemiology; see [4, 11]. The use of nomograms in clinical decision making includes prediction of prostate cancer severity [12, 13], and the Partin nomogram (known as Partin Table) [14] which has been used mainly as a prognostic tool over the last two decades for the prediction of pathological stage in prostate cancer.

More recently, nomograms have been used to visualise statistical models. A nomogram generated from a model plays the role of a graphical ‘predict’ function facilitating the calculation of a point estimate of the response variable for a particular set of values of the explanatory variables [15, 16]. It consists of rulers for each predictor (*X*) along with two rulers to convert the total points to the desired risk scale. The length of the i^th^ predictor ruler provides a visual representation of the relative effect sizes *β*) of each explanatory variable in the model. It is defined in the Eq 1, which is calculated as a scaled version of the proportion of the predictor’s contribution divided by the maximum predictor contribution:

$$\begin{array}{c} \frac{range\left(X_{i}\right)*\beta_{i}}{\max_{i}\left[range\left(X_{i}\right)*\beta_{i}\right]} \end{array}$$ (1)

Software is available to create nomograms for statistical models in SAS [17], Stata [18], Python [19] and as online tools for constructing simple JAVA-based interactive nomograms [20] as well as the rms [21] and hdnom [22] packages in **R**.

The rms package in **R** [21] includes the nomogram function to generate nomograms from a fitted statistical model. For example, the code below creates a nomogram from a logistic regression model used to model the relationships between age, gender and passenger class on surviving the Titanic disaster (data are included in the PASWR package [23]).

R> data(titanic3)

R> t.data <- datadist(titanic3)

R> options(datadist = “t.data”)

R> fit <- lrm(survived ~ age + pclass + sex, data = titanic3)

R> plot(nomogram(fit, fun = function(x)plogis(x)))

**Fig 2:**
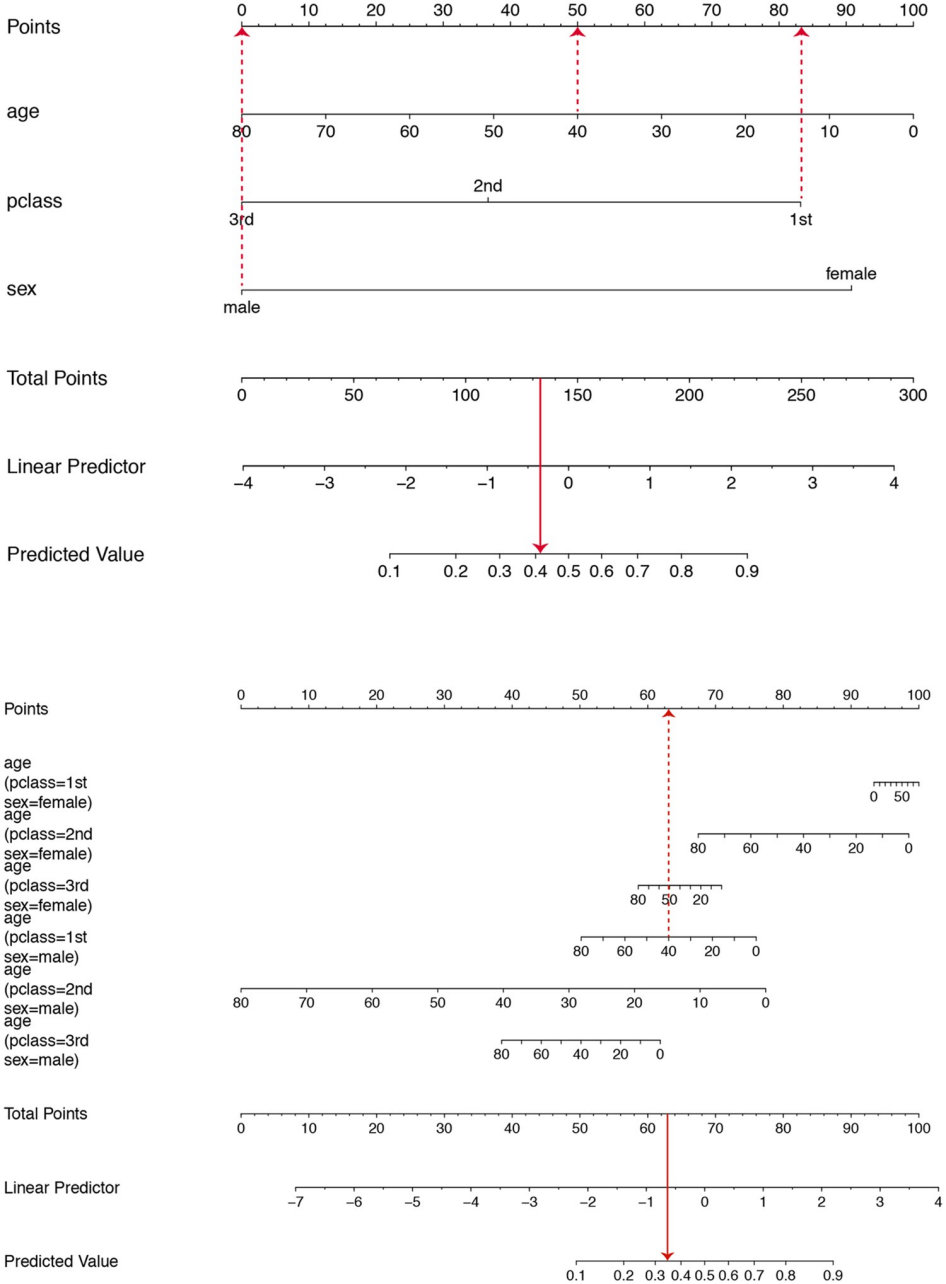
Nomograms for calculating the probability of surviving the Titanic disaster using the rms package. A logistic regression model containing age, sex and passenger class as main effects is displayed on top while a model including all their two-way interactions is shown at the bottom.

The resulting nomogram is given in Fig 2. The horizontal line at the top labelled ‘points’ allows the effect size of each variable to be assessed, including direct visualisation of the potential variation in the effect. The points measure the contribution of the values/levels of the explanatory variable of interest on the ‘linear predictor’ scale. The total points are then mapped from the ‘linear predictor’ to obtain the ‘predicted value’, which in this case is a probability. It is clear from the graph on the top that all three explanatory variables make similar contributions to the linear predictor, over the spread of value/levels that each can take. For example, in the main effects model presented the effect of gender on the probability of survival is comparable to a change in the age of 70 years. Such an insight is less evident from the estimated coefficients. The model represented at the bottom in Fig 2 incorporates higher order interactions and suggests that the effect of age is most prominent in 2^nd^ class males. As an example, the superimposed dashed lines correspond to a 40-year-old male passenger with a 1^st^ class ticket. The estimated probability of survival is calculated as 0.41 based on the main effects model and 0.35 when all two-way interactions are included.

**Fig 3:**
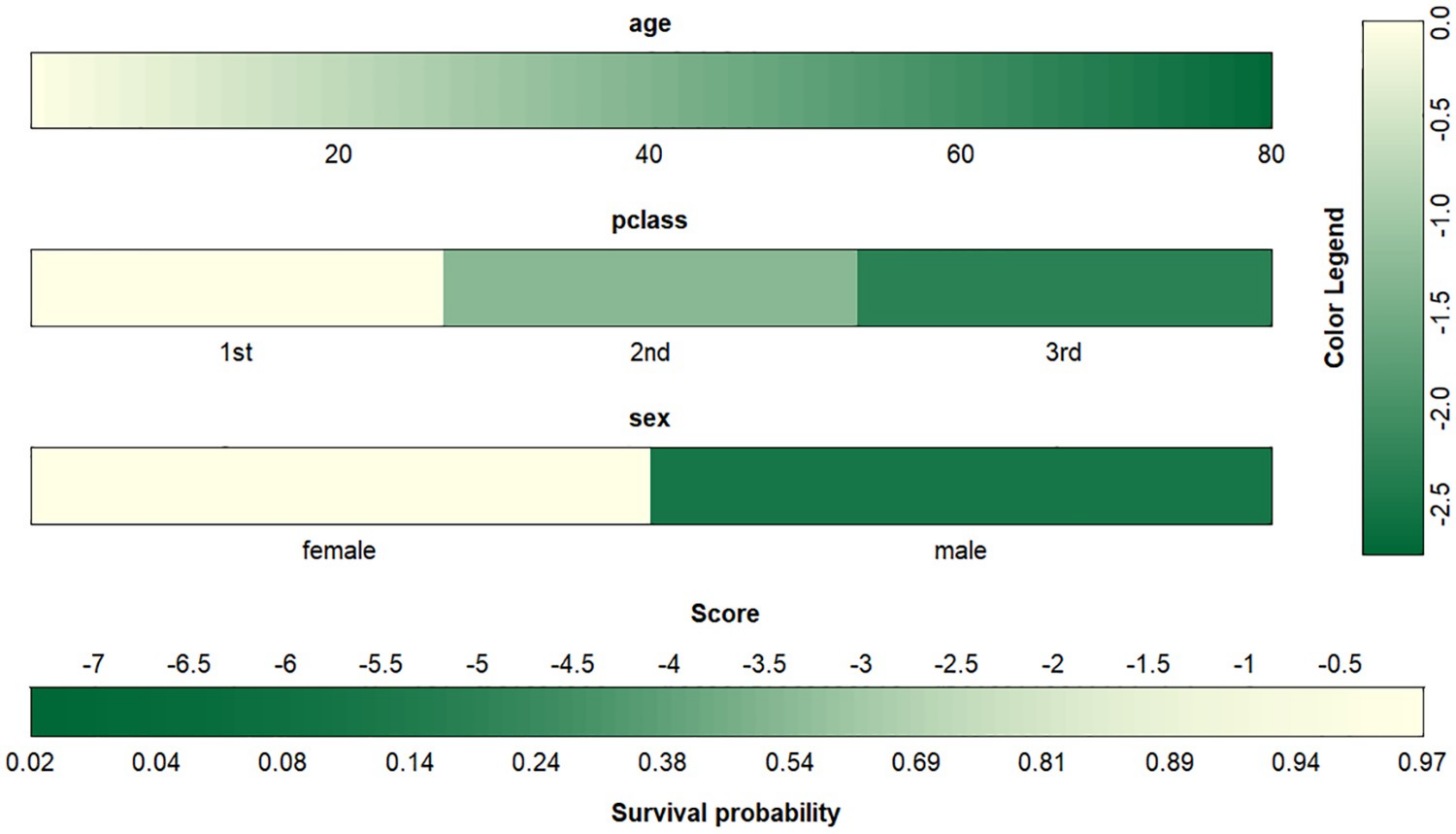
Colour-based nomogram representing the probability of surviving the Titanic disaster in the main effect model using the VRPM package.

More recent developments in this area include the coloured-based nomogram in the VRPM package [24] proposed by Belle and Caster in 2015 [25]. Fig 3 represents a colour-based nomogram for the main effect model fitted to the Titanic data. The survival probability is calculated by summing up the contribution from each covariate/factor when matched to the colour legend. The effect of gender relative to age is again evident. The accuracy of any prediction depends on the users’ ability to match colours accordingly.

When modelling a binary outcome (e.g. using logistic regression), modelling the log odds is mathematically attractive, summarising a treatment effect as an odds ratio may be misleading [26, 27]. A nomogram is helpful as the predicted response is given as a probability.

It has been argued [28] that a summary quoting the underlying probabilities is more informative than one based on ratios of odds or probabilities. Odds and probability are often (mistakenly) used interchangeably where large and small odds ratios are interpreted as meaningful without reference to the underline probability of the event of interest. Similarly, in a logistic regression model the importance of an explanatory variable is often assessed (incorrectly) by the magnitude of the regression coefficient while ignoring the scale of the variable (e.g. hours, minutes, days) or by the p-value while ignoring the sample size. Given that the response variable of interest is a binary outcome, the effect of each variable on the response may be more easily translated if represented on a probabilistic scale.

Nomograms achieve this and provide an attractive and useful graphical summary of a logistic regression model with the ability to make predictions as probabilities. Static Nomograms can become cumbersome to use as models become more complex (e.g. higher order interactions) or the inclusion of smoothers [29], and when visualising uncertainty (e.g. interval estimates). One solution is to create dynamic nomograms as interactive applications to visualise statistical models such that effect sizes can be assessed graphically, by visualising the predicted value and corresponding confidence interval automatically.

## Dynamic nomograms

The emergence of the rpanel [30] and Shiny packages [31] for **R** allowed the creation of interactive, user-friendly graphical interfaces to be displayed either in a standalone window or delivered as a webpage.

The DynNom package [32] is built to generate dynamic nomograms as a Shiny application for a variety of statistical models to allow a reader to interact with the model in a user-friendly manner. The package can be install from the CRAN repository using the following command:

R> install.packages(“DynNom”)

For example, the **R** code below will create a dynamic nomogram, using a simple function, for a logistic regression model of the Titanic data.

R> fit <- glm(survived ~ (age + pclass + sex) ^ 3, titanic3, family = “binomial”)

R> DynNom(fit)

**Fig 4:**
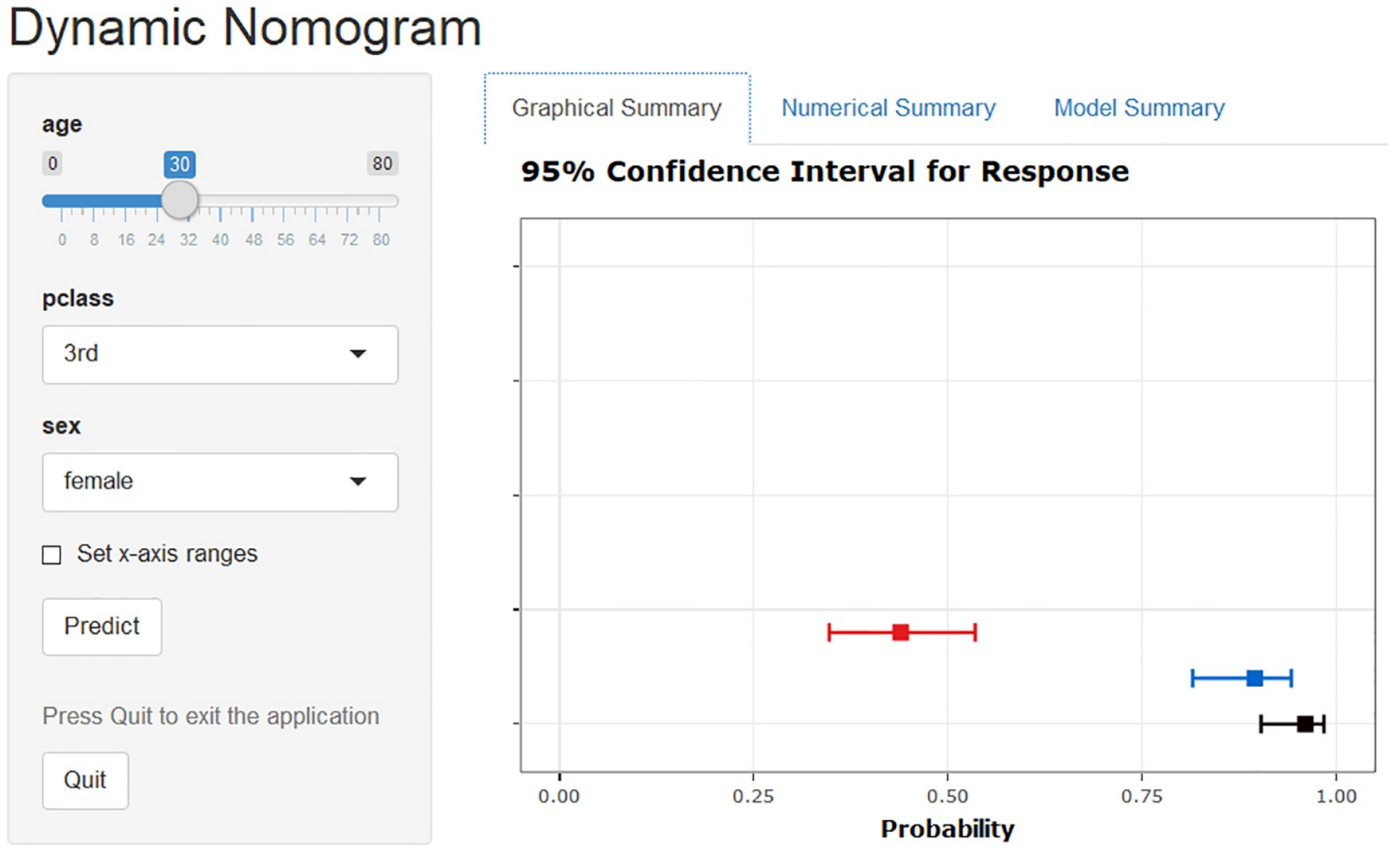
Dynamic nomogram of the logistic model with all three-way interactions fitted to the Titanic data using DynNom function. The plot represents survival probability (with 95% confidence interval) of 30-year-old females with different ticket classes. The actual explanatory values and their corresponding predictions are given in the ‘Numerical Summary’ tab in S1 Fig, and the ‘Model Summary’ tab is provided in S2 Fig.

The dynamic nomogram displays sliders for covariates (bounded by their observed ranges) and drop-down boxes for factors. The predict function maps the appropriate inverse link function (*η* = *g*(*μ*)) from the generalised linear model object to generate predicted values (on the scale of the response variable) and corresponding 95% confidence intervals (see Fig 4). The predicted value and corresponding confidence interval are plotted and presented in the tooltip labels and the ‘Numerical Summary’ tab. Further, a formatted model output summary is displayed in the ‘Model Summary’ tab.

Predicted quantities are accompanied by measures of uncertainty [33] in the form of confidence intervals. The confidence interval for the mean response will quantify the uncertainty around the estimation of the mean response for a given set of explanatory variables. In generalised linear model models, for example, an approximate 100(1 − *α*)% confidence interval for the mean response for a given new observation ($x_{*}^{T}=\left(1,x_{1},x_{2},...,x_{p}\right)$) is calculated as:

$$\begin{array}{c} {\hat{\eta }}_{i}\pm u_{1-\frac{\alpha }{2}}\sqrt{x_{*}^{T}\hat{\Sigma }x_{*}} \end{array}$$ (2)

where *x* is a vector of observed predictors, ${\hat{\eta }}_{i}=x_{*}^{T}\hat{\beta }$ is the linear equation estimates, $u_{1-\frac{\alpha }{2}}$ indicates the corresponding critical value and Σ denotes the covariance matrix. The confidence interval is estimated in the linear form; however, for a clear interpretation, it is best to apply the inverse link transformation *g*^−1^(.) (see Table 1) to the confidence limits in formula (2) [34].

**Table 1:** Commonly used link functions.

Density	Link function(name)	*μ* = *g*^−1^(*η*)
Normal	*η* = *μ* (identity)	*η*
Binomial	$\eta =log(\frac{\mu }{1-\mu })$ (logit)	$\frac{exp(\eta )}{\left(1+exp(\eta )\right)}$
Poisson	*η* = *log*(*μ*) (logarithm)	*exp*(*η*)
Gamma	*η* = −*μ*^−1^ (reciprocal)	$\frac{1}{\eta }$

Two main functions of the package are DynNom and DNbuilder functions. The former extracts the model class from the fitted model to build a dynamic nomogram as a shiny app which computes and displays the point and interval estimate using Eq 2 while the latter generates the files necessary to deploy the app.

The DynNom package supports model objects created by the lm, glm and coxph functions in **R**. It also supports ols, Glm, lrm and cph models in the rms package which allow the inclusion of smoothing splines as well as gam functions from mgcv [35] and gam packages [36]. When applied to a Cox proportional hazards model object, the estimated survival function will be displayed with alpha-blending (i.e. thickness of the line reflex the number of subjects over time) to represent the underlying uncertainty of the estimates. Table 2 summarises all the model objects supported by DynNom which cover a wide variety of the most common models fitted depending on the type of response variable of interest.

**Table 2:** R model objects (package) supported in DynNom.

Response variable		Model	R functions (package)
Discrete			
	Binary	Logistic regression	glm (stats), lrm (rms)
	Count data	Poisson regression	glm (stats), Glm (rms)
Continuous			
	Normal	Linear regression	lm (stats), ols (rms)
	Positive	Gamma regression	glm (stats), Glm (rms)
	Time-to-event	Cox proportional hazards	coxph (survival), cph (rms)
		Generalized additive model	gam (mgcv), gam (gam)

## Examples

For illustration, dynamic nomograms for a Poisson response, a linear regression incorporating smoothing splines and a Cox proportional hazards model for time-to-event data are presented in the following sections. Examples involving Gamma regression and generalized additive model objects are not presented for brevity as the same procedure applies.

### Modelling a count response

The ‘crabs’ dataset [37] (available in the glm2 package [38]) is used as an example of visualising a Poisson regression model. This dataset comprises 173 female horseshoe crabs, each of which had at least one male crab attached to it in their nest. This study aims to investigate factors affecting the number of male partners in addition to the female’s primary partner (called satellites). The explanatory variables include the width of the female (Width) and a binary factor indicating whether the female colouring is dark or not (Dark). A Poisson regression model has been used previously [37] to model the relationship between the ‘Width’ and ‘Dark’ on the number of partners. The summary of the fitted Poisson model is given in Table 3, where the effect of Width and Dark are identified as significant (at the 5% level).

**Table 3:** Model summary for the crabs data illustration.

	Estimate	Std. Error	z-value	p-value
Intercept	-2.8202	0.5707	-4.94	<0.0001
Width	0.1492	0.0207	7.20	<0.0001
Dark (yes)	-0.2652	0.1024	-2.59	0.0096

**Fig 5:**
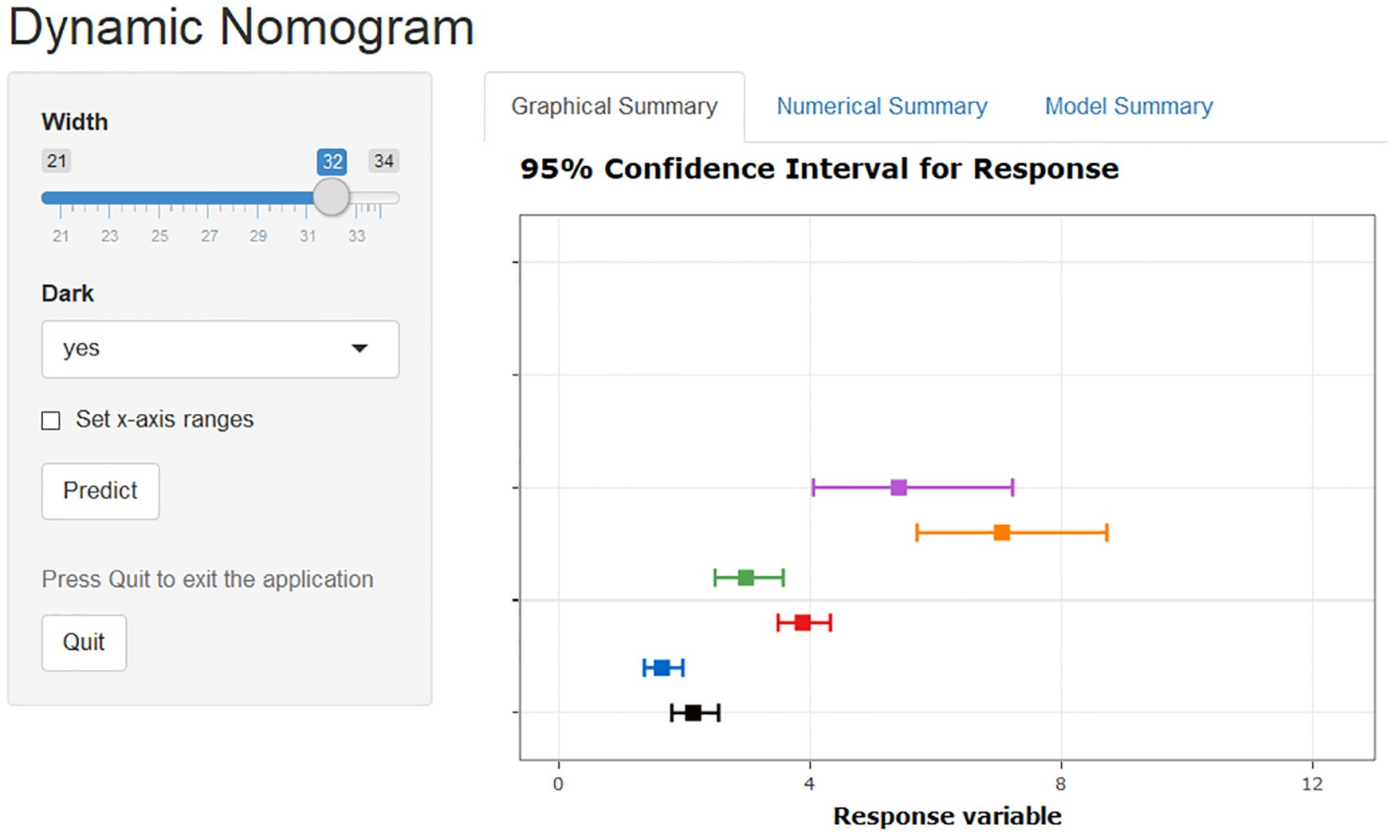
Dynamic nomogram for a Poisson regression model fitted to the crabs data. The plot displays the predicted number of additional male partners of female crabs of 24, 28 or 32 centimetres width of either dark or light colour. The actual explanatory values/levels and predictions are given in the ‘Numerical Summary’ tab in S3 Fig.

The accompanying dynamic nomogram (Fig 5) can be created as follows:

R> fit2 <- glm(Satellites ~ Width + Dark, family = poisson(log), data = crabs)

R> DynNom(fit2)

The estimates displayed in Fig 5 are for the predicted (mean) response for scenarios covering the two levels of crab colour and three values of Width. The joint effects of ‘Width’ and ‘Dark’ (and corresponding uncertainty estimates) on the response are visually evident which would be more difficult to glean from the model summary.

### Modelling a continuous response with a smoothing spline

**Fig 6:**
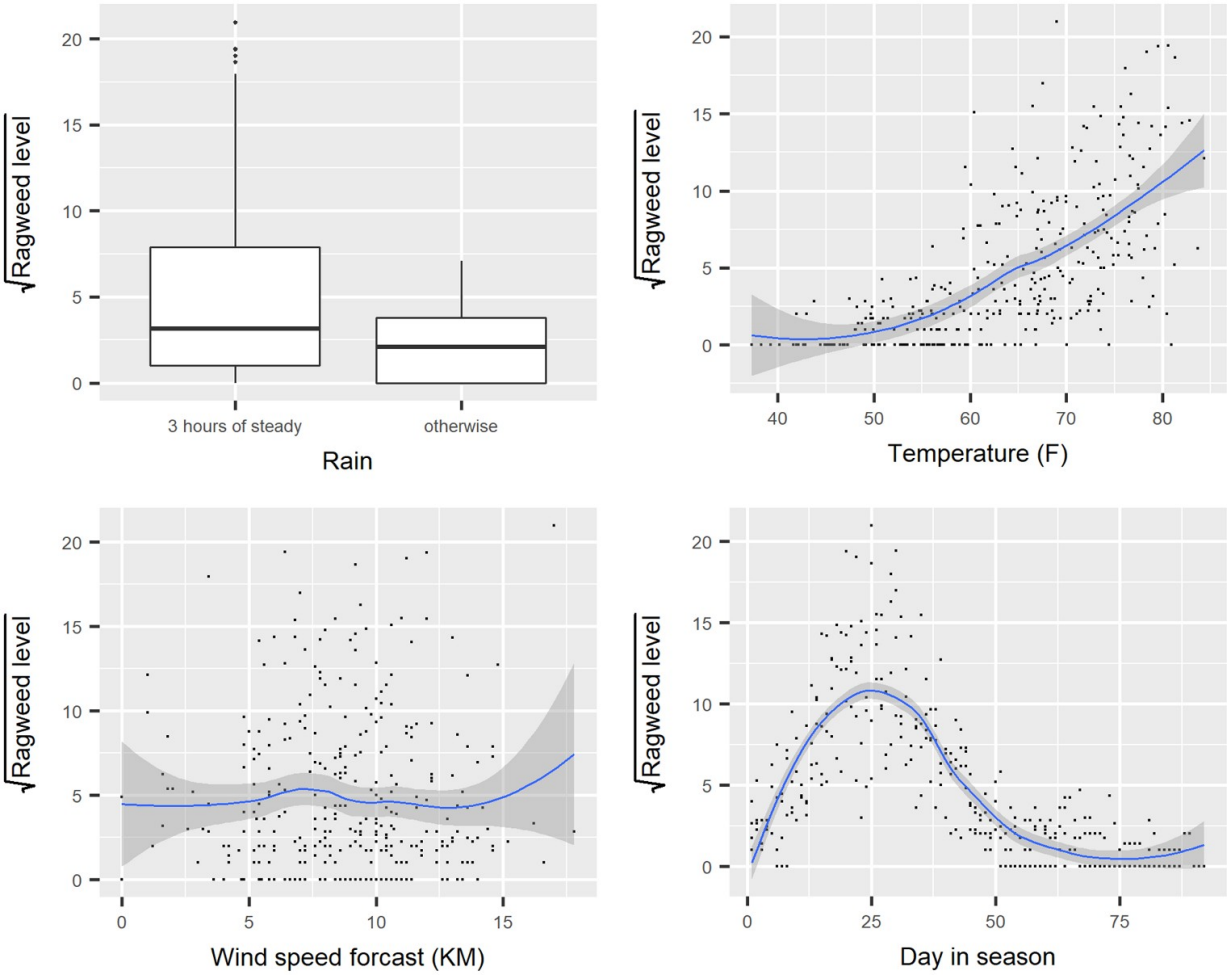
Relationships between the square root of ragweed level and the other variables.

Interpreting the role explanatory variables play can be difficult in a model when the functional form of an explanatory is modelled as a smooth function. For example, the ‘ragweed’ dataset, introduced in [39] is an example where a regression model can be used for a continuous response incorporating a smoothing spline (data is available in the SemiPar package [40]). The aim is to forecast the ragweed pollen level as a function of the temperature, rain, wind speed forecast for the following day and pollen season day. The corresponding boxplot and the scatterplot (Fig 6) illustrate the marginal relationships between the explanatory variables and the (transformed) response, the square root of ragweed level.

**Fig 7:**
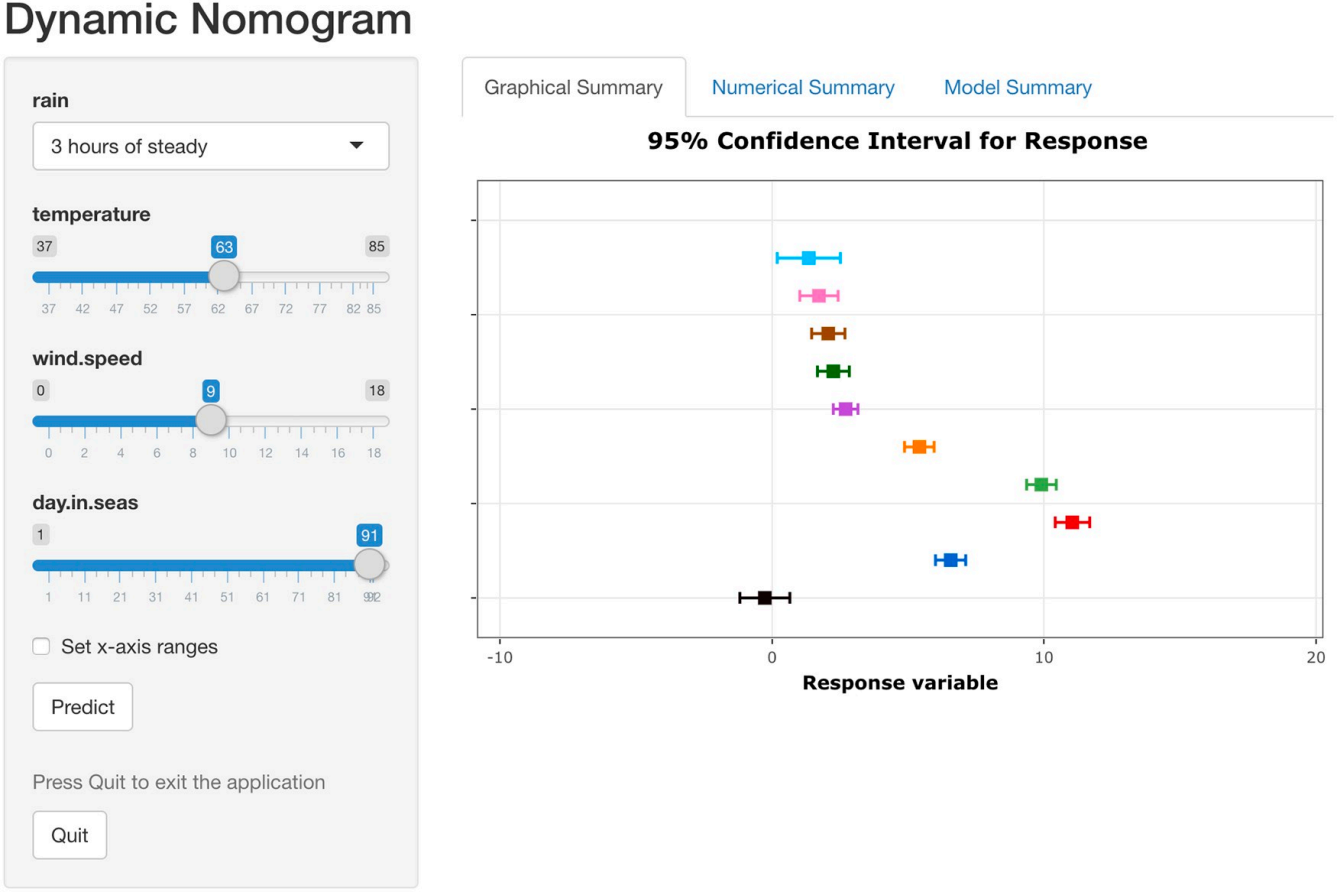
Dynamic nomogram for a regression model with a smoothing spline fitted to the ragweed data. The plot displays the predicted response for ‘day in season’ set at 1, 11, 21, 31, 41, 51, 61, 71, 81 and 91 respectively while fixing all other explanatory variables at the mean/mode as appropriate. The actual explanatory values/levels and predictions are given in the ‘Numerical Summary’ tab in S4 Fig.

The nonlinear relationship between the day in pollen season and the mean response is modelled using a restricted cubic spline function, with temperature, and wind speed as (linear) predictors and rain presence as a factor. The following code fits the model using the ols function in the rms package (Table 4) and creates the dynamic nomogram shown in Fig 7.

R> ragweed$sqrtragweed <- sqrt(ragweed$ragweed)

R> dd <- datadist(ragweed)

R> options(datadist = “dd”)

R> fit3 <- ols(sqrtragweed ~ rain + temperature + wind.speed + rcs(day.in.seas, 5), data = ragweed)

R> DynNom(fit3)

**Table 4:** Model summary for the ragweed data illustration. The ′, ″, ‴, ‴′ represent different knot effects in the restricted cubic splines specified for pollen season day.

	Estimate	Std. Error	t-value	p-value
Intercept	-9.4656	1.4014	-6.75	<0.0001
rain(otherwise)	-1.3593	0.4031	-3.37	0.0008
temperature	0.1003	0.0184	5.45	<0.0001
wind.speed	0.2431	0.0392	6.20	<0.0001
day.in.seas′	0.6967	0.0352	19.78	<0.0001
day.in.seas″	-3.8046	0.2059	-18.48	<0.0001
day.in.seas‴	9.3518	0.5999	15.59	<0.0001
day.in.seas‴′	-7.8311	0.8159	-9.60	<0.0001

The need for a non-linear function for the day in season variable appears apparent where the effect increases for up to 25 days in the season and then decreases from that time point onwards. Interpreting the effect of this covariate using the model summary alone, while adjusting for rain, temperature and wind speed, is less clear.

The effect of ‘days in season’ is arguably more accessible using a dynamic nomogram (Fig 7) by choosing values of this covariate ranging from low to high and setting the remaining explanatory variables at their mode (for factors) and means (for covariates).

### Modelling a time-to-event response

Classical graphical summaries for time-to-event data include plots of the estimated survival or hazard functions. When modelling the effect of covariates and factors on time-to-event data, the Cox proportional hazards model is a popular choice as the underlying survival distribution does not need to be specified. The interpretation of the effect of explanatory variables on the ‘risk’ of the event of interest is often made using hazard ratios or by using a plot of the estimated survival function conditioning on the set of explanatory variables of interest. For example, the lung cancer dataset in the survival package [41] includes the time to death for 228 advanced lung cancer patients where gender, age, weight loss and daily activity performance scores (such as ECOG) were recorded as potentially useful explanatory variables. The code needed to fit a Cox proportional hazards model and the corresponding dynamic nomogram to investigate the effect of these explanatory variables on time to death is displayed below.

R> model <- coxph(Surv(time, status) ~ age + wt.loss + ph.ecog + sex, data = lung)

R> DynNom(model)

The summary of the fitted Cox proportional hazards model is given in Table 5, where the effect of each explanatory variable is given on the log scale and as a hazard ratio.

**Table 5:** The Cox proportional hazards model summary for the lung data illustration.

	Estimate	Std. Error	HRatio	95% CI	p-value
age	0.0134	0.0096	1.01	(0.99, 1.03)	0.1650
ph.ecog	0.5151	0.1260	1.67	(1.31, 2.14)	<0.0001
wt.loss	-0.0090	0.0067	0.99	(0.98, 1.00)	0.1761
sex(female)	-0.5908	0.1753	0.55	(0.39, 0.78)	0.0008

**Fig 8:**
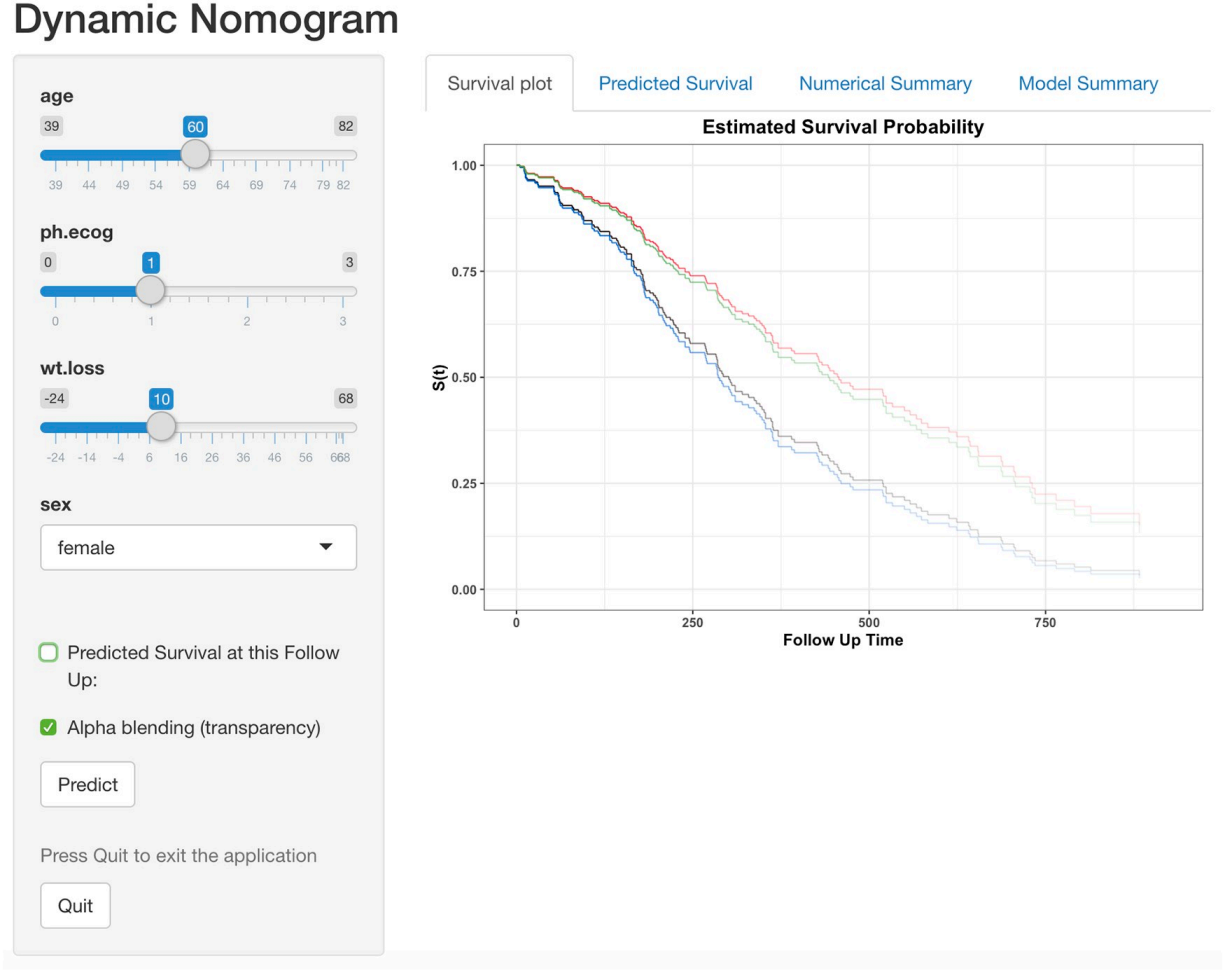
Dynamic nomogram for the Cox proportional hazards model fitted to the lung cancer data. The Kaplan-Meier plot displays survival curves correspond to 55 and 60 years old males/females (ECOG and weight loss set at their mean). The predicted survival time (with 95% confidence interval) at 250 days is given in the ‘Predicted survival’ tab (S5 Fig) and the actual explanatory values/levels and predictions are given in the ‘Numerical Summary’ tab (S6 Fig).

The dynamic nomogram (Fig 8) presents the estimated survival function for a given set of values of the explanatory values which avoid the use of hazard ratios and interpretation of effects that act multiplicatively. Rather than clutter the graph by including the corresponding interval estimates for each survival function, the degree of uncertainty in the estimate is highlighted graphically by adjusting the colour transparency (alpha-blending) to represent the number of subjects at risk over time. If required the predicted survival at any given follow-up time point can be generated using the ‘Predicted Survival’ tab.

## Publishing dynamic nomograms

In order to make a dynamic nomogram accessible to a wider audience additional functions (i.e. DNbuilder family functions) are provided to generate the files necessary (i.e. ui.r, server.r and global.r) to publish the dynamic nomogram as illustrated below:

R> library(PASWR)

R> library(DynNom)

R> data(titanic3)

R> fit <- glm(survived ~ (age + pclass + sex) ^ 3, titanic3, family = “binomial”)

R> DNbuilder(fit)

The corresponding files can then be uploaded to a server running Shiny (e.g. https://www.shinyapps.io/) which facilitates the deployment of Shiny applications. For example, the dynamic nomogram built for the analysis of the Titanic data described above is published at https://dynnom.shinyapps.io/titanic/. Examples of the use of the DynNom package (and the DNbuilder function in particular) to generate linked dynamic nomograms to statistical models published in research articles includes [42–47].

All models presented in the literature could in theory and practice have an accompanying web address to direct the reader to the corresponding dynamic nomogram allowing them to interact with the model and identify the exact nature of the model fitted. The ‘Model Summary’ tab provides the (native) model summary from **R** allowing an examination of the precise model fitted and accompanying model summaries (e.g. *R*^2^, *AIC* etc.), which makes it clear as to the actual model (and its parametrisation) fitted.

## Conclusion

As inferential statistical methods become more computational, the models arising are increasingly complex and are often hard to interpret and translate, in particular for non-statisticians. Dynamic nomograms are one such translational tool to help address this issue. They provide a visual representation of a statistical model where the importance of each explanatory variable, in terms of their effect size, becomes more evident through the assessment of the change in the predicted value. This is particularly useful for models containing modifiable risk factors.

The ability to accompany a model summary in a journal with a link to the corresponding dynamic nomogram has the potential to make models more translatable, and analyses more transparent in terms of fulfilling the recommendations of making all research understandable and reproducible.

## Supporting information

S1 Fig The ‘Numerical Summary’ tab of the dynamic nomogram in Fig 4. (TIF)

S2 Fig The ‘Model Summary’ tab of the dynamic nomogram in Fig 4. (TIF)

S3 Fig The ‘Numerical Summary’ tab of the dynamic nomogram in Fig 5. (TIF)

S4 Fig The ‘Numerical Summary’ tab of the dynamic nomogram in Fig 7. (TIF)

S5 Fig The ‘Predicted survival’ tab of the dynamic nomogram in Fig 8. (TIF)

S6 Fig The ‘Numerical Summary’ tab of the dynamic nomogram in Fig 8. (TIF)
