# Supplementary figures and images for: Visualising statistical models using dynamic nomograms
Source: PLoS One. 2019 Nov 15;14(11):e0225253. doi: 10.1371/journal.pone.0225253 (PMC6857916; doi:10.1371/journal.pone.0225253)

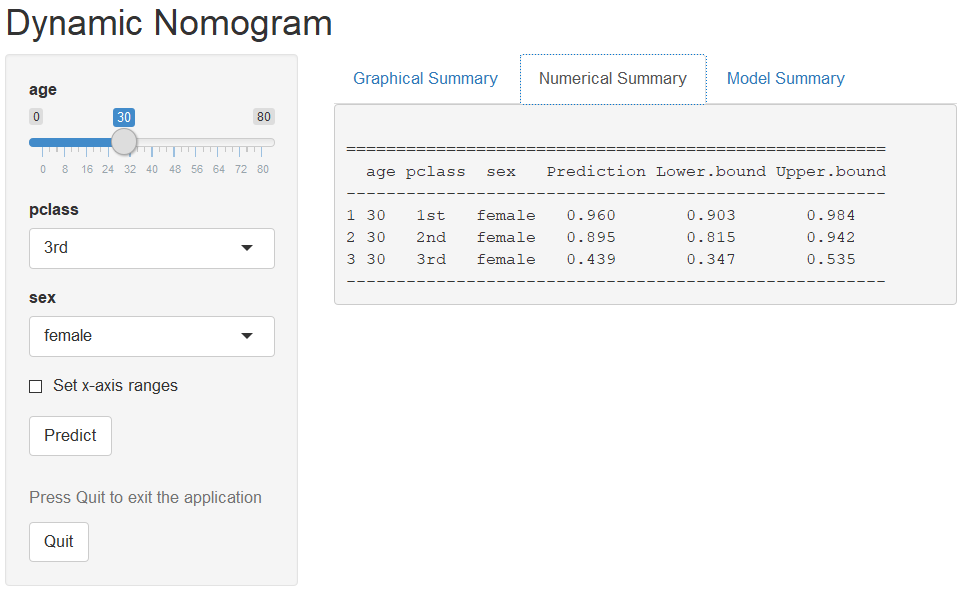

Supplement: S1 Fig — (TIF) [file pone.0225253.s001.tif]

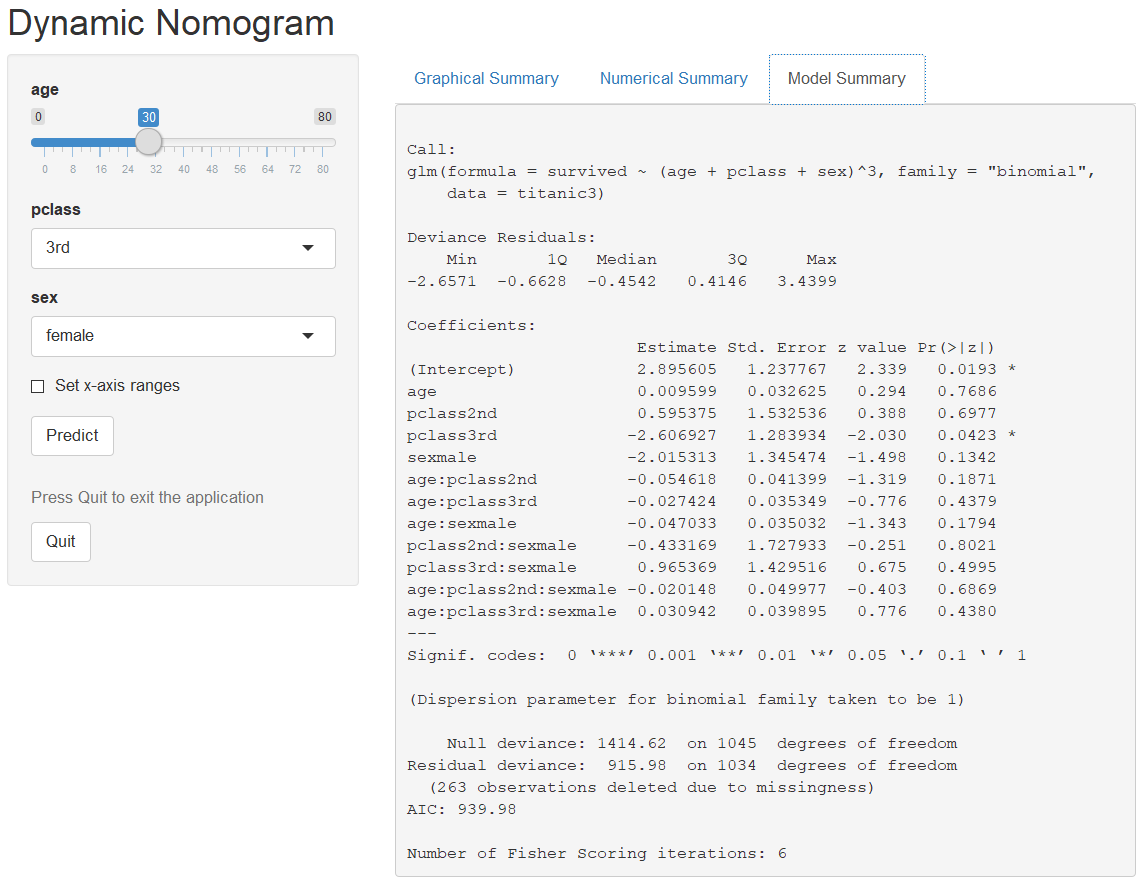

Supplement: S2 Fig — (TIF) [file pone.0225253.s002.tif]

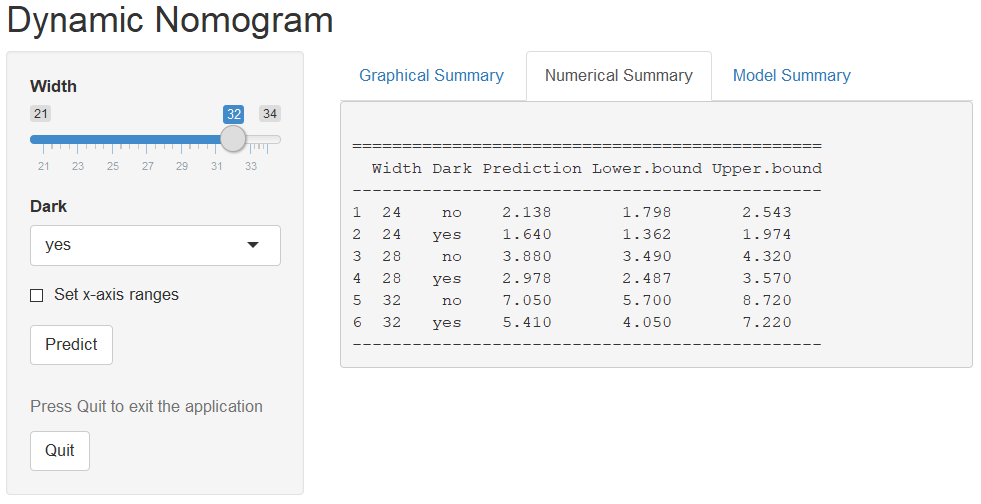

Supplement: S3 Fig — (TIF) [file pone.0225253.s003.tif]

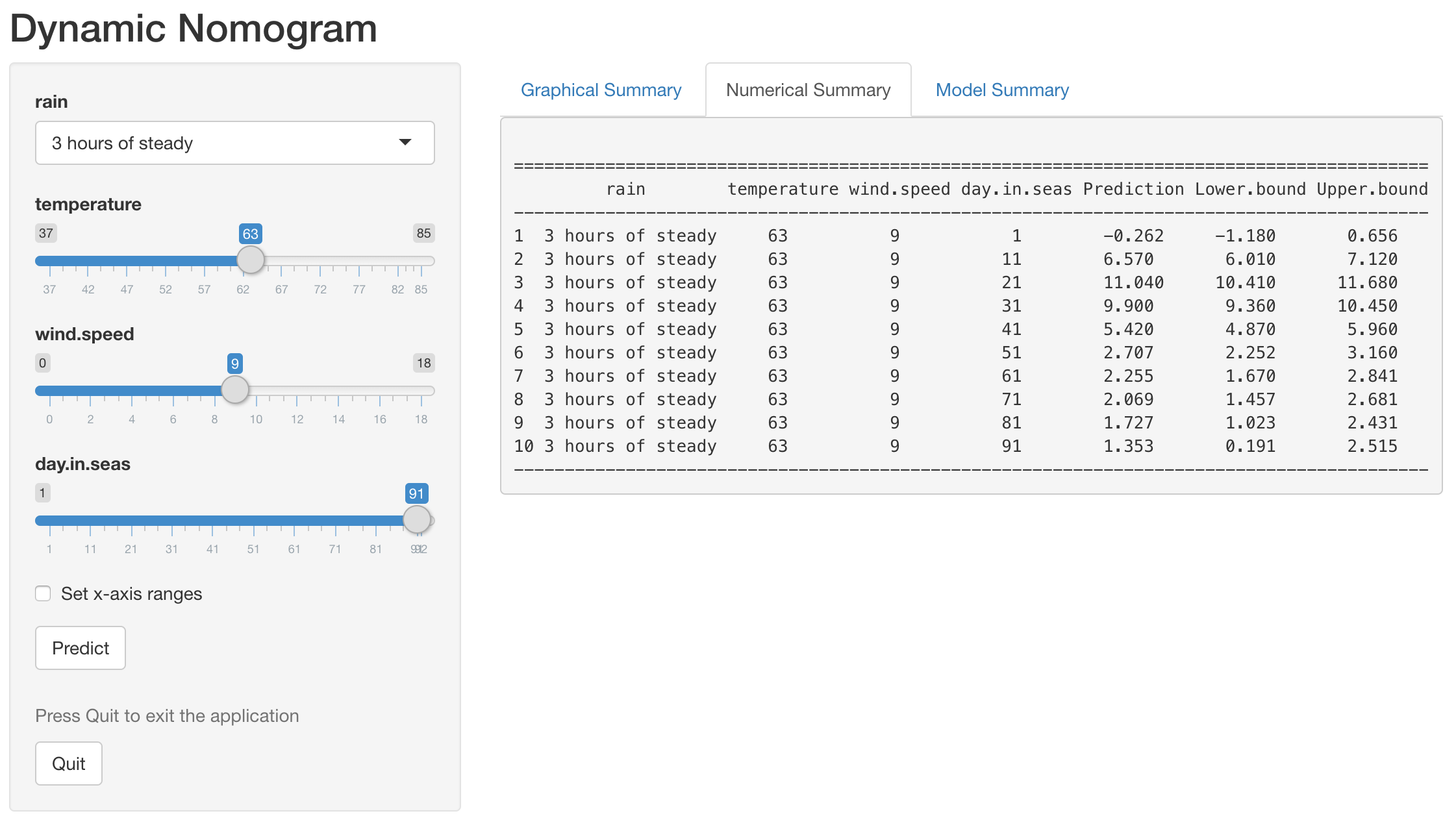

Supplement: S4 Fig — (TIF) [file pone.0225253.s004.tif]

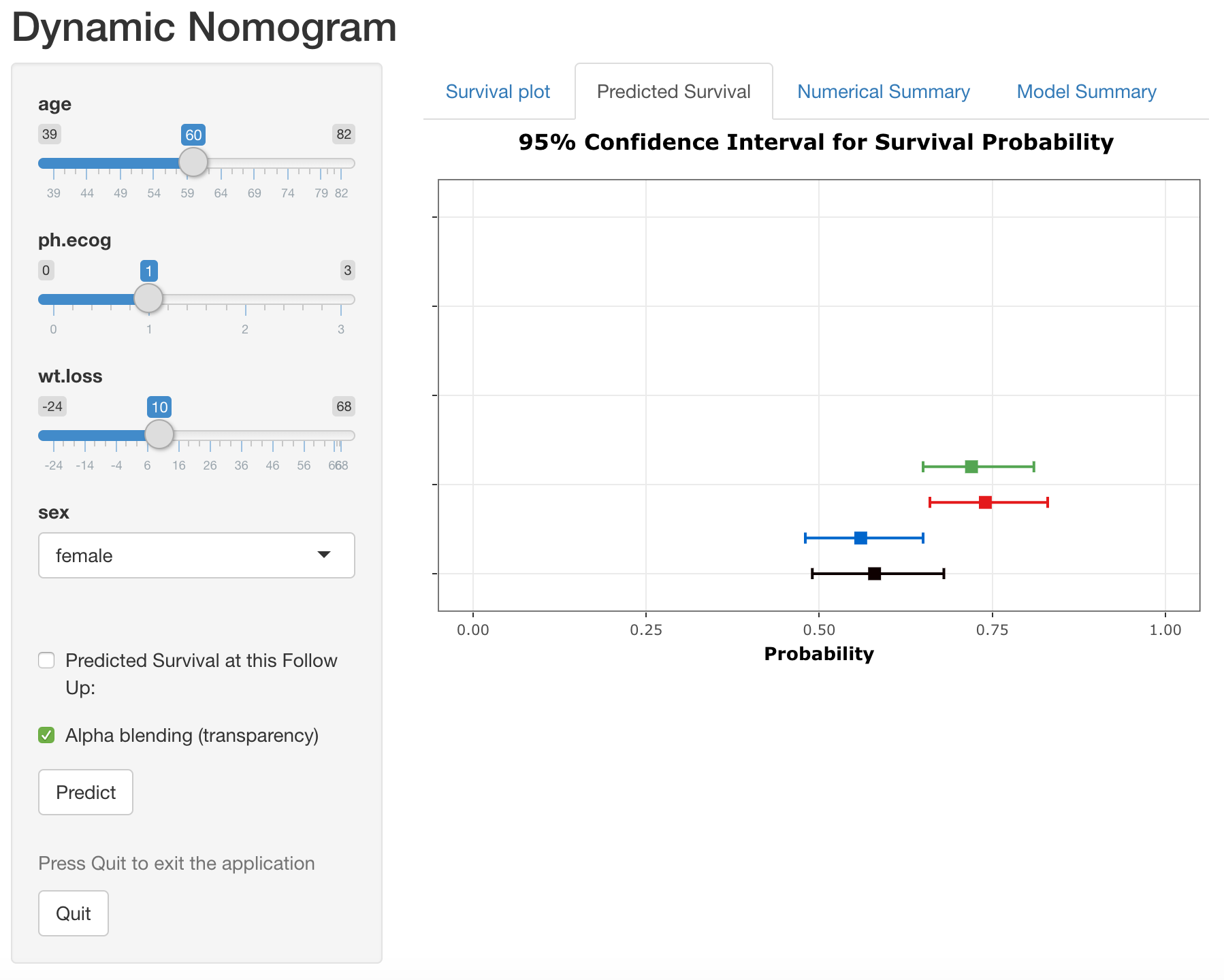

Supplement: S5 Fig — (TIF) [file pone.0225253.s005.tif]

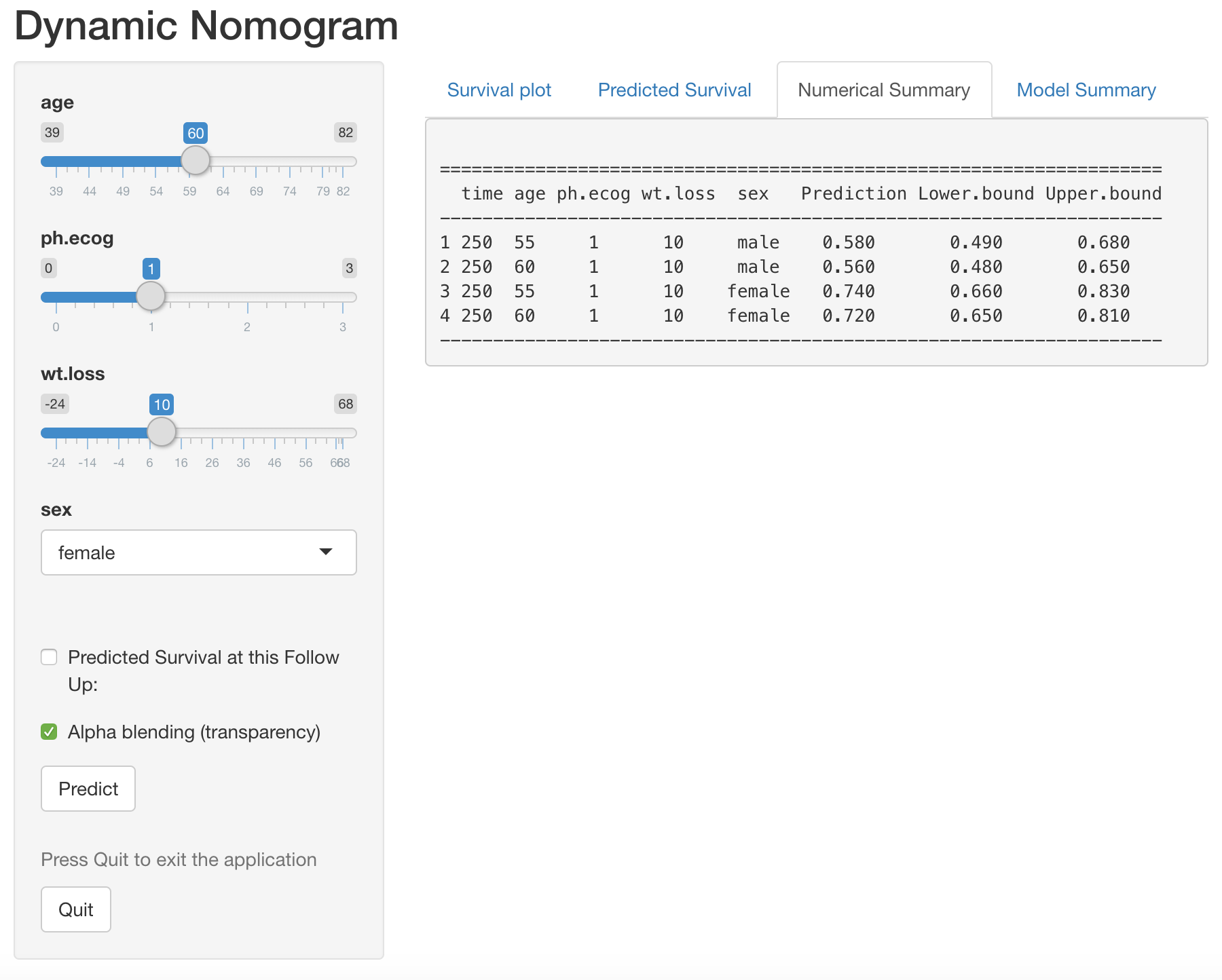

Supplement: S6 Fig — (TIF) [file pone.0225253.s006.tif]
